# Vitamin D and Calcium Supplement Use and High-Risk Breast Cancer: A Case–Control Study among *BRCA1* and *BRCA2* Mutation Carriers

**DOI:** 10.3390/cancers15102790

**Published:** 2023-05-17

**Authors:** Emma Guyonnet, Shana J. Kim, Katherine Pullella, Cindy X. W. Zhang, Jeanna M. McCuaig, Susan Armel, Steven A. Narod, Joanne Kotsopoulos

**Affiliations:** 1 Women’s College Research Institute, Women’s College Hospital, Toronto, ON M5G 1N8, Canadakatherine.pullella@wchospital.ca (K.P.);; 2 Department of Nutritional Sciences, University of Toronto, Toronto, ON M5S 1A8, Canada; 3 Dalla Lana School of Public Health, University of Toronto, Toronto, ON M5T 3M7, Canada; 4 Department of Physiology, University of Toronto, Toronto, ON M5S 1A8, Canada; 5 Department of Molecular Genetics, University of Toronto, Toronto, ON M5S 1A8, Canada; 6 Familial Cancer Clinic, Princess Margaret Hospital Cancer Centre, University Health Network, Toronto, ON M5G 2M9, Canada; 7 Department of Medicine, University of Toronto, Toronto, ON M5S 3H2, Canada

**Keywords:** vitamin D, calcium, supplements, *BRCA*, breast cancer

## Abstract

**Simple Summary:**

The relationship between vitamin D and calcium supplement use and breast cancer among women in the general population is not clear. Furthermore, whether such an association exists among women at high risk due to a *BRCA1* or *BRCA2* mutation has not been investigated. Thus, we evaluated the association between vitamin D and/or calcium supplement use and breast cancer in this high-risk population. In this case–control study, we identified 134 women diagnosed with breast cancer and 276 women without breast cancer. Women with a *BRCA* mutation who consumed vitamin D-containing supplements had 46% lower odds of having breast cancer compared to those who did not take any supplements. Increasing vitamin D and calcium supplement intake was inversely associated with the odds of having breast cancer. Higher vitamin D and/or calcium supplement intake may be associated with lower breast cancer risk in this high-risk population.

**Abstract:**

The role of vitamin D and calcium use in the development of breast cancer among women in the general population is not clear. Furthermore, whether vitamin D and calcium supplement use are associated with breast cancer in high-risk populations has not been evaluated. Thus, we evaluated the association between vitamin D and/or calcium supplement use and breast cancer among women with a pathogenic variant (mutation) in *BRCA1* or *BRCA2*. *BRCA* mutation carriers enrolled in a longitudinal study were invited to complete a supplemental questionnaire on lifetime supplement use. Cases included women with a prevalent diagnosis of invasive breast cancer, and controls had no history of breast cancer. Vitamin D and calcium use were categorized as never/ever use, and as tertiles of supplement intake (total average daily supplement use). Unconditional logistic regression was used to estimate the odds ratio (OR) and 95% confidence intervals (CIs) of breast cancer. This study included 134 breast cancer cases and 276 controls. Women who used vitamin D-containing supplements had 46% lower odds of having breast cancer compared to those who never used supplements (OR 0.54; 95% CI 0.31, 0.91; *p* = 0.02). Increasing vitamin D and calcium supplement intake was inversely associated with the odds of having breast cancer (*p*-trend = 0.04). Findings were suggestively stronger among BRCA1 mutation carriers; however, analyses were limited by small strata. These findings suggest a potential inverse association between vitamin D and calcium supplementation and *BRCA* breast cancer. Additional studies are warranted to confirm these findings and accurately inform clinical care guidelines.

## 1. Introduction

The lifetime risk for developing breast cancer among Canadian women is 12% [1]; however, women who carry a pathogenic or likely pathogenic (‘mutation’ hereafter) in the tumor suppressor genes *BRCA1* or *BRCA2* have an elevated risk estimated at 70% by age 80 [2]. Current management options for these high-risk women are limited to primary prevention with bilateral mastectomy or intensified screening with annual MRI and mammography aimed at early detection [3]. Vitamin supplement use is very popular among Canadian women [4], but the extent to which consuming vitamin D supplements may affect breast cancer risk in *BRCA1* or *BRCA2* mutation carriers is not known.

Findings from studies investigating the role of vitamin D use on breast cancer risk among women in the general population have been inconsistent. In a recent meta-analysis of observational data (*n* = 22 studies), Hossain et al. reported that supplemental vitamin D was inversely associated with the risk of developing breast cancer and that the protective effect was stronger at doses greater than 10 mcg/day [5,6]. In contrast, a recent international pooling project showed that circulating vitamin D was not associated with subsequent breast cancer incidence, and randomized controlled trials have similarly failed to report an association between intake of vitamin D and risk [7,8].

To our knowledge, there are no other studies to date that have evaluated the association between vitamin D supplement use and the risk of breast cancer among *BRCA1* or *BRCA2* mutation carriers. Given the high rates of supplement use among women in Canada and the United States, as well as the emerging evidence that vitamin D plays a role in *BRCA1*-mediated cancers [4,9,10], we conducted a case–control study to investigate the association between supplemental vitamin D and breast cancer among women with a *BRCA1* or *BRCA2* mutation enrolled in an ongoing longitudinal study of hereditary cancer. Given that vitamin D is typically supplemented with calcium, we also investigated the association between supplemental calcium use and breast cancer, as well as the interactive relationship between vitamin D and calcium supplement use and breast cancer. 

## 2. Materials and Methods

### 2.1. Study Population

Eligible participants were identified from an ongoing longitudinal study of *BRCA* mutation carriers initiated in 1995 and previously described in detail [11,12]. Women aged 18–70 years with a pathogenic or likely pathogenic variant (i.e., mutation) in *BRCA1* and/or *BRCA2* were eligible for inclusion. Various techniques were used to detect mutations, but direct DNA sequencing confirmed all nucleotide sequences. The current study included prevalent breast cancer cases and controls from ten participating centers across seven provinces in Canada. Institutional review boards of the host institutions approved the study, and all study subjects gave written informed consent to participate.

### 2.2. Data Collection

All women completed a *baseline* questionnaire at the time of enrollment from 1994 to 2014 either by mail or by phone with a research assistant or genetic counselor. These questionnaires collected detailed information on family and personal histories, including preventive surgery, medication use, screening practices, and cancer diagnoses. This questionnaire also included questions on lifestyle factors, such as weight (kg) and drinking habits (never/ever). Between September 2014 and September 2016, participants from the 10 Canadian centers were invited to complete a *supplemental* questionnaire, by mail or over the phone, which collected detailed information on past and current supplement use from the age of 18. Women were asked to recall the type of supplements used, their brand names, frequency of use per week, dosage, and duration of use. Information on supplements taken during pregnancy was also collected.

### 2.3. Assessment of Supplement Use

The current study focused on vitamin D and calcium use. Supplement use was assessed in two different ways: (1) *vitamin D-containing supplement use*, categorized as never/ever using any supplements that contained vitamin D alone and in combination with other nutrients, such as in multivitamins or prenatal supplements, and (2) *total average daily vitamin D supplement use* (mcg/day), calculated from all supplements that contained vitamin D. For example, total average daily vitamin D supplement use was calculated from vitamin D-specific supplements, multivitamins, and prenatal supplements that contained vitamin D as follows:

$$\sum \mathrm{dose}\;\mathrm{of}\;\mathrm{vitamin}\;D\;\mathrm{per}\;\mathrm{pill}\times \;\frac{\mathrm{frequency}\;\mathrm{of}\;\mathrm{use}\;\mathrm{per}\;\mathrm{week}}{7\;\mathrm{days}\;a\;\mathrm{week}}\;\times \frac{\mathrm{years}\;\mathrm{of}\;\mathrm{supplement}\;\mathrm{used}}{\mathrm{total}\;\mathrm{study}\;\mathrm{years}}$$

The same approach was used to determine calcium-containing supplement use (never/ever) and total average daily calcium supplement use (mg/day).

If the brand names of the supplements were given, but doses of vitamin D or calcium were missing, doses were assigned based on the published formulation for the specific brand. If both brand names and doses were missing (*n* = 65), default doses were assigned based on the formulation of the Jamieson brand (25 mcg for vitamin D and 500 mg for calcium). Multivitamins with missing brand names and doses (*n* = 97) were also assigned to reflect the formulation of the Jamieson brand (15 mcg of vitamin D and 175 mg of calcium). Prenatal supplements with missing doses and brand names (*n* = 33) had doses assigned from the formulation of the prenatal supplement brand Materna (15 mcg of vitamin D and 250 mg of calcium). Jamieson and Materna were used as they are the most popular brands in Canada. Observations were removed if the information on the years or frequency of use was missing (*n* = 118 for total average daily vitamin D use, *n* = 110 for total average daily calcium use). When compared with manufacturers’ labels, the validity of self-reported supplement use and dose had previously been shown to be good for multivitamins, vitamin D, and calcium (kappa 0.58–0.78) [13]. Detailed information on dietary intake was not collected in the longitudinal study.

### 2.4. Assessment of Case and Control Status

The disease was self-reported by the participants in the study questionnaire. Case subjects were women with a prevalent diagnosis of invasive breast cancer. Control subjects were women who never had a history of breast cancer. Medical records were requested for all women who reported having breast cancer and pathology was confirmed in 50% of cases.

### 2.5. Subject Selection

**Figure 1:**
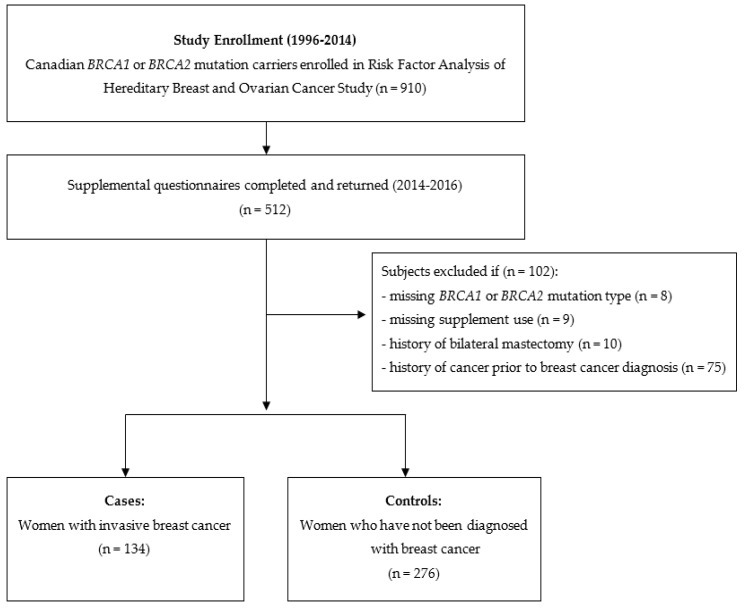
Flow diagram describing subject selection.

Figure 1 outlines the subject selection for the current study. Women were eligible for inclusion in this analysis if they had completed both the baseline and supplemental questionnaires. As previously described [12], among the 910 participants who were invited to participate in the study, 512 completed the supplemental questionnaire. Participants were excluded if they had a bilateral mastectomy (*n* = 10), a history of cancer before their breast cancer diagnosis (*n* = 75), were missing supplement information (*n* = 9), and mutation type (*n* = 8). After exclusions, there were 410 women included in the study, including 134 cases and 276 controls.

### 2.6. Statistical Analysis

A case–control analysis was used to evaluate the association between vitamin D or calcium supplement use and breast cancer. Among the cases, all exposures and covariates were censored one year prior to the diagnosis of breast cancer. We stratified vitamin D and calcium supplement use as never or ever use and divided the total average daily vitamin D and calcium supplement use into tertiles (never, moderate, high) based on the distribution of supplement intake of the entire cohort.

We used Student’s *t*-test and a chi-square test to evaluate the differences between the baseline continuous and categorical variables between the cases and controls, respectively. Unconditional logistic regression was used to estimate the odds ratio (OR) and 95% confidence intervals (CIs) for breast cancer associated with supplement use. We constructed a basic model adjusting for age (continuous) and *BRCA1* or *BRCA2* mutation type. The multivariable model included additional covariates, such as menopausal status (premenopausal or postmenopausal), body mass index (BMI) (continuous), parity (0, 1–2, ≥3 live births), alcohol consumption (never/ever), and smoking status (never/ever). Subjects who had a missing BMI (*n* = 7) were assigned the median BMI of the entire sample. Those with missing smoking (*n* = 2) or alcohol (*n* = 2) values were assigned the mode. One subject with a mutation in both genes was classified as a *BRCA1* mutation carrier. The *P* for trend was estimated by modeling the trend per unit increase in vitamin D (mcg) and calcium (mg) to assess dose–response relationships. Analyses were further stratified by *BRCA1* or *BRCA2* mutation type to assess the impact of supplement use among each population.

In a secondary analysis, we performed a joint effects analysis to assess the multiplicative interaction between vitamin D and calcium supplement use and breast cancer. The aim of the joint effects analysis was to observe if the combined effects of vitamin D and calcium are larger than the sum of their individual effects.

A quantitative bias analysis was conducted to quantify the level of bias in our study that may be attributed to possible exposure misclassification of vitamin intake (recall bias) and/or the presence of unmeasured confounding. Multidimensional bias analysis tested a range of sensitivity and specificity values for exposure misclassification ranging from 0.1 to 0.9, which quantifies the level of misclassification needed to attenuate the study estimate to null [14]. Furthermore, an E-value was calculated to determine the minimal strength of association needed for unmeasured confounders to explain away the study estimate to null [15].

All statistical analyses were conducted using SAS OnDemand for Academics (SAS Institute, Cary, NC, USA). All *p*-values were two-sided and considered statistically significant if *p* ≤ 0.05.

## 3. Results

### 3.1. Subject Characteristics

Table 1 summarizes the characteristics of the 410 study participants included in the current analysis by case or control status. Case subjects had a higher BMI than controls (26.4 kg/m^2^ vs. 24.9 kg/m^2^; *p* = 0.01) and were less likely to be nulliparous compared to controls (17.9% vs. 32.6%, *p* = 0.0004). A higher proportion of cases than controls were premenopausal (78.1% vs. 65.6%, *p* = 0.01). Cases were less likely to have used *vitamin D-specific* supplements than controls (7.7% vs. 15.0% for vitamin D; *p* = 0.05). Case subjects also had lower intakes of *total average daily vitamin D* and *calcium supplements* than controls (2.5 mcg/day vs. 4.6 mcg/day for vitamin D; *p* = 0.01 and 36.9 mg/day vs. 65.6 mg/day for calcium; *p* = 0.02).

### 3.2. Vitamin D Supplement Use and Breast Cancer

The association between vitamin D-containing supplement use and breast cancer among *BRCA1* and *BRCA2* mutation carriers is presented in Table 2. Women who used vitamin D-containing supplements had 46% lower odds of having breast cancer compared to those who never used supplements (multivariate OR = 0.54; 95% CI 0.31, 0.91; *p* = 0.02). Women with higher total daily vitamin D supplement use (>7.50 mcg/day) had 56% lower odds of breast cancer compared to those with the lowest tertile of intake (multivariate OR = 0.44; 95% CI 0.22, 0.89; *p*-trend = 0.04). In the analyses stratified by *BRCA* mutations, these associations were stronger among women with a *BRCA1* mutation, although the number of women in the *BRCA2* subgroup was few (Table 2).

### 3.3. Calcium Supplement Use and Breast Cancer

There was a suggestive, albeit not statistically significant, inverse association between the use of any calcium-containing supplement and breast cancer (multivariate OR = 0.61; 95% CI 0.36, 1.05; *p* = 0.07); however, increasing calcium supplement intake was associated with lower odds of having breast cancer (multivariate OR = 0.56; 95% CI 0.28, 1.09; *p*-trend  =  0.04) (Table 3). In the stratified analyses, the inverse associations were significant for *BRCA1* but not *BRCA2* mutation carriers; however, analyses were limited by small numbers in the subgroups (Table 3).

### 3.4. Joint Effects of Vitamin D and Calcium and Breast Cancer

We also evaluated the joint effects of both calcium and vitamin D supplements (Table 4). Compared to the individuals who never used either supplement, the OR of breast cancer among individuals who used both was 0.60 (95% CI 0.34, 1.03; *p* = 0.07).

### 3.5. Quantitative Bias Analysis

The multidimensional bias analysis tested a range of values for the sensitivity and specificity of vitamin D supplement use to quantify the level of measurement bias. Assuming a non-differential misclassification of the exposure due to recall bias, the corrected OR only exceeded 1 when the sensitivity was <0.6 and specificity was <0.3. Assuming a differential misclassification of the exposure due to recall bias between cases and controls, the corrected OR only exceeded 1 when the sensitivity and specificity were <0.6, or when the sensitivity and specificity were >0.6 and higher in the controls compared to the cases. In all situations, it is unlikely that exposure misclassification would have a large enough effect to reverse the direction of our findings, as previous studies demonstrated the validity of self-reported supplement use is good, and we would expect higher sensitivity/specificity of exposure measurement among cases compared to controls in differential recall bias [16].

The E-value calculated for the primary outcome was 2.06. Therefore, the observed association between vitamin D-containing supplement use and breast cancer could only be explained away by an unmeasured confounder with a minimum association of an OR of 2.06.

## 4. Discussion

In this study, we found that women at high risk of developing breast cancer due to an inherited *BRCA1* or *BRCA2* mutation who used vitamin D supplements had 46% lower odds of having breast cancer. Increasing vitamin D supplement intake was also inversely associated with the odds of having breast cancer. Although there was a suggestive, albeit not statistically significant, inverse association between calcium supplement use and breast cancer, we observed a linear dose–response relationship between calcium supplement intake and breast cancer. The effects were stronger for women with a *BRCA1* mutation; however, this was based on small strata. A joint effects analysis did not suggest multiplicative combined effects of vitamin D and calcium supplement use and breast cancer in our study. While recall bias and unmeasured confounding is possible in this retrospective study, quantitative bias analysis suggests these biases are unlikely to reverse the direction of our observed effects. Despite the relatively small sample size, these results provide preliminary evidence of an inverse association between high vitamin D and calcium supplement use and breast cancer among women with a *BRCA* mutation.

We observed a linear dose–response trend between vitamin D supplement intake and breast cancer. Our findings are in alignment with observational studies conducted among women in the general population [5,6]. Estébanez et al. reported an inverse association between vitamin D supplement use and breast cancer risk in a meta-analysis of five case–control studies (OR 0.78; 95% CI 0.63, 0.98) [16]. Importantly, in a cohort of 50,884 high-risk women (1611 incident cases) who had a sister with breast cancer but had not had breast cancer themselves, vitamin D supplement use was associated with a decreased risk of breast cancer over five years of follow-up (Hazard Ratio (HR) 0.89; 95% CI 0.81, 0.99) [17]. However, a meta-analysis of seven clinical trials concluded no significant effect of vitamin D supplementation on breast cancer risk [8]. Specifically, the Vitamin D and Omega-3 Trial (VITAL) trial with 25,871 participants and 246 incident cases showed that vitamin D supplementation at a dose of 50 mcg/day over a follow-up of 5.3 years did not reduce invasive breast cancer risk (HR 1.02; 95% CI 0.79, 1.31), while Keum and Giovannucci reported that the benefits of vitamin D supplementation over 2–7 years may be limited to cancer mortality only [18,19]. Due to the small number of incident cases and relatively short trial durations, these trials were likely not sufficiently powered to detect the effect on risk, which may explain the discrepant findings.

Although increasing calcium supplement intake was associated with lower odds of having breast cancer in our study, the main source of calcium was from multivitamins and not calcium-specific supplements alone. Since most multivitamins also contain vitamin D, it is possible this association between calcium supplements and breast cancer may be attributed to either vitamin D or the combined use of both supplements. Although limited by a small sample size, this was supported by our joint analysis that showed a stronger inverse association for those who used vitamin D supplements only and combined vitamin D and calcium than for those who used calcium supplements only. In a meta-analysis of ten randomized controlled trials, including seven studies on breast cancer, ≥500 mg/day of calcium supplements without the co-administration of vitamin D did not affect breast cancer risk (relative risk (RR) 1.01; 95% CI 0.64, 1.59, *p* = 0.97) [20]. In contrast, a reanalysis of the Women’s Health Initiative trial data showed calcium and vitamin D-containing supplements decreased the risk of invasive breast cancer (HR 0.80; 95% CI 0.66, 0.96; *p* = 0.02) [21]. Since calcium was administered with vitamin D, it was not possible to detect the individual effects on breast cancer risk, but the null findings from the meta-analysis suggest calcium’s beneficial effect may be linked to the co-administration of vitamin D [20]. This was further supported by the lack of multiplicative effects of vitamin D and calcium supplement use observed in our joint effects analysis.

Emerging evidence suggests vitamin D and calcium may protect against breast cancer by inducing anti-proliferative and pro-apoptotic effects; however, their effect on the development of *BRCA*-associated breast cancer is unclear. Vitamin D first binds to and activates the vitamin D receptor (VDR) expressed in mammary glands. This activation leads to the inhibition of cell proliferation by suppressing growth signals and activating growth-inhibitory signals [22,23,24,25]. Grotsky et al. suggest that vitamin D prevents the degradation of tumor protein p53-binding protein 1 (TP53BP1) in cancers mediated by a *BRCA1* mutation, therefore increasing plasma vitamin D levels and inhibiting cell proliferation in *BRCA1* mutation carriers [10]. A recent study also reported higher VDR expression, associated with prolonged overall survival, in *BRCA1*-mutated breast cancers compared to sporadic breast cancers [26]. Furthermore, VDR-targeted agonists, such as calcitriol, seemed to inhibit the proliferation of triple-negative and VDR-positive breast cancer cells, supporting the tumor-suppressing effect of VDR [27]. However, studies on *BRCA2* mutations are lacking. Multiple other mechanisms linked to apoptosis have been suggested. One study suggests apoptosis is triggered by BCL2 family proteins, known to regulate cell death [22]. Peterlik et al. propose that vitamin D induces apoptosis by stimulating calcium release from intracellular stores, resulting in an increase in calcium in the cytosol, which then triggers caspase-independent programmed cell death [28]. Even though little is known about the calcium-sensing receptor, its activation increases the influx of calcium across the membrane in breast cancer cells [28]. This rise in intracellular calcium may induce pro-apoptotic signals, similar to those caused by vitamin D [28].

There were various limitations with the current study, notably the small sample size, particularly in the stratified analyses. Only 56% of women responded to the supplemental questionnaire, which could have contributed to selection bias. The inclusion of prevalent cases may contribute to survival bias, which is difficult to account for. Given the retrospective nature, data collection from women following a breast cancer diagnosis may have introduced recall bias in some cases. Nevertheless, our multidimensional bias analysis demonstrated that the non-differential and differential recall bias of supplement intake is unlikely to reverse the direction of the study estimates. There also remains the possibility of unmeasured confounding in the study; however, the E-value of 2.06 demonstrates that unmeasured confounding is unlikely to explain the study estimates away to null, and the analysis accounted for the most important confounders. Finally, we were not able to disentangle the effects of vitamin D and calcium alone since supplement use mostly consisted of multivitamins rather than individual supplements; however, we were able to evaluate the total average daily intake of each supplement and breast cancer risk. Lastly, we only analyzed supplemental vitamin D and calcium use and not total intake (i.e., dietary and supplement use), which likely underestimated the participants’ vitamin D and calcium intake.

## 5. Conclusions

To our knowledge, this represents the first evaluation of vitamin D and calcium supplement use and breast cancer risk specifically in this high-risk population. Our findings provide preliminary evidence of an inverse association between both exposures and the risk of breast cancer, particularly among *BRCA1* mutation carriers. Despite this protective effect, women with a *BRCA1* or *BRCA2* mutation should not consume more than the recommended daily intake of these supplements. It will be important to continue to clarify these associations, especially in prospective analyses using plasma vitamin D and calcium levels, biomarkers that reflect total intake from both dietary sources and supplement use.

## Figures and Tables

**Table 1:** A comparison of the case and control of *BRCA1* or *BRCA2* mutation carriers.

Characteristic	Cases	Controls	*p* ^a^
	(*n* = 134)	(*n* = 276)	
Age at breast cancer diagnosis (year)	41.5 ± 8.3 ^b^		
Age (year) ^c^	41.5 ± 8.3	42.7 ± 12.2	0.24
Mutation (%)			0.15
*BRCA1*	61.9	54.3	
*BRCA2*	38.1	45.7	
BMI (kg/m^2^)	26.4 ± 5.7	24.9 ± 4.7	0.01
Age at menarche (year)	12.4 ± 1.4	12.6 ± 1.5	0.13
Menopausal status (%)			0.01
Premenopausal	78.1	65.6	
Postmenopausal	21.9	34.4	
Oral contraceptive use			0.42
Never	15.6	19.0	
Ever	84.4	81.0	
Parity (%) ^d^			0.0004
0	17.9	32.6	
1–2	61.9	41.7	
≥3	20.2	25.7	
Prophylactic bilateral oophorectomy (%)	6.8	12.0	0.11
Alcohol consumption (%)			0.16
Never	25.4	19.3	
Ever	74.6	80.7	
Smoking status (%)			0.07
Never	55.2	64.5	
Ever	44.8	35.5	
Multivitamin supplement use (%)			0.12
Never	76.9	69.2	
Ever	23.1	30.8	
Prenatal supplement use (%) ^e^			0.16
Never	54.1	45.7	
Ever	45.9	54.3	
Vitamin D			
Vitamin D-specific supplement use (%)	7.7	15.0	0.05
Vitamin D-containing supplement use (%)	60.2	68.5	0.11
Total average daily vitamin D supplement use (mcg/day)	2.5 ± 5.0	4.6 ± 8.4	0.01
Calcium			
Calcium-specific supplement use (%)	3.4	8.7	0.06
Calcium-containing supplement use (%)	60.3	66.5	0.25
Total average daily calcium supplement use (mg/day)	36.9 ± 75.9	65.6 ± 126.6	0.02

^a^ *p*-values were calculated using Student’s *t*-test for continuous variables and a chi-square test for categorical variables. ^b^ All such values are mean ± standard deviation (SD). ^c^ Age at breast cancer diagnosis for cases or year of baseline questionnaire for controls. ^d^ Parity: number of live full-term births. ^e^ Only among women with a history of pregnancy (except if ending in abortion), *n* = 297.

**Table 2:** OR and 95% CI of breast cancer by vitamin D supplement use, stratified by *BRCA1* or *BRCA2* mutation type.

	Cases/Controls	OR (95% CI) Basic Model ^a^	*p*	OR (95% CI) Multivariable Model ^b^	*p*
Any vitamin D-containing supplement use ^c^					
*All Participants*					
Never	47/79	Ref.	Ref.	Ref.	Ref.
Ever	71/172	0.70 (0.44, 1.10)	0.12	0.54 (0.31, 0.91)	0.02
*BRCA1*					
Never	30/41	Ref.	Ref.	Ref.	Ref.
Ever	41/94	0.60 (0.33, 1.10)	0.10	0.40 (0.20, 0.81)	0.01
*BRCA2*					
Never	17/38	Ref.	Ref.	Ref.	Ref.
Ever	30/78	0.86 (0.42, 1.75)	0.67	0.79 (0.35, 1.78)	0.57
Total average daily vitamin D supplement use (mcg/day) ^c^					
*All Participants*					
None	46/79	Ref.	Ref.	Ref.	Ref.
1.07 ≤ 7.50	26/44	1.03 (0.56, 1.90)	0.91	0.72 (0.35, 1.46)	0.36
>7.50	22/75	0.51 (0.28, 0.92)	0.03	0.44 (0.22, 0.89)	0.02
*p*-trend per mcg increase in vitamin D			0.03		0.04
*BRCA1*					
None	29/41	Ref.	Ref.	Ref.	Ref.
1.07 ≤ 7.50	17/20	1.19 (0.53, 2.65)	0.68	0.74 (0.30, 1.86)	0.53
>7.50	11/42	0.37 (0.16, 0.84)	0.02	0.28 (0.11, 0.71)	0.007
*p*-trend per mcg increase in vitamin D			0.03		0.01
*BRCA2*					
None	17/38	Ref.	Ref.	Ref.	Ref.
1.07 ≤ 7.50	9/24	0.87 (0.33, 2.27)	0.77	0.66 (0.22, 1.97)	0.46
>7.50	11/33	0.75 (0.31, 1.84)	0.53	0.82 (0.29, 2.27)	0.70
*p*-trend per mcg increase in vitamin D			0.41		0.63

Unconditional logistic regression was used to estimate the OR and 95% CI. ^a^ Adjusted for age (continuous) and *BRCA1* or *BRCA2* mutation type. ^b^ Adjusted for age (continuous), *BRCA1* or *BRCA2* mutation type, menopausal status (premenopausal or postmenopausal), BMI (continuous), parity (0, 1–2, ≥3), alcohol consumption (ever or never), and smoking status (ever or never). ^c^ Includes vitamin D from sources such as multivitamins, prenatal supplements, and vitamin D-specific supplements.

**Table 3:** OR and 95% CI of breast cancer by calcium supplement use, stratified by *BRCA1* or *BRCA2* mutation type.

	Cases/Controls	OR (95% CI) Basic Model ^a^	*p*	OR (95% CI) Multivariable Model ^b^	*p*
Any calcium-containing supplement use ^c^					
*All Participants*					
Never	46/86	Ref.	Ref.	Ref.	Ref.
Ever	70/171	0.77 (0.49, 1.22)	0.27	0.61 (0.36, 1.05)	0.07
*BRCA1*					
Never	32/44	Ref.	Ref.	Ref.	Ref.
Ever	41/95	0.60 (0.33, 1.08)	0.09	0.41 (0.21, 0.81)	0.01
*BRCA2*					
Never	14/42	Ref.	Ref.	Ref.	Ref.
Ever	29/76	1.15 (0.55, 2.41)	0.72	1.13 (0.49, 2.63)	0.77
Total average daily calcium supplement use (mg/day) ^c^					
*All Participants*					
None	45/86	Ref.	Ref.	Ref.	Ref.
15.63 ≤ 105.88	24/45	1.02 (0.55, 1.90)	0.94	0.71 (0.35, 1.44)	0.34
>105.88	24/76	0.61 (0.34, 1.09)	0.09	0.56 (0.28, 1.09)	0.09
*p*-trend per mg increase in calcium			0.05		0.04
*BRCA1*					
None	31/44	Ref.	Ref.	Ref.	Ref.
15.63 ≤ 105.88	14/24	0.81 (0.36, 1.81)	0.60	0.50 (0.20, 1.25)	0.14
>105.88	14/39	0.50 (0.23, 1.08)	0.08	0.38 (0.16, 0.91)	0.03
*p*-trend per mg increase in calcium			0.08		0.05
*BRCA2*					
None	14/42	Ref.	Ref.	Ref.	Ref.
15.63 ≤ 105.88	10/21	1.47 (0.56, 3.86)	0.44	1.17 (0.39, 3.48)	0.78
>105.88	10/37	0.80 (0.32, 2.03)	0.64	1.00 (0.35, 2.84)	0.99
*p*-trend per mg increase in calcium			0.31		0.54

Unconditional logistic regression was used to estimate the OR and 95% CI. ^a^ Adjusted for age (continuous) and *BRCA1* or *BRCA2* mutation type. ^b^ Adjusted for age (continuous), *BRCA1* or *BRCA2* mutation type, menopausal status (premenopausal or postmenopausal), BMI (continuous), parity (0, 1–2, ≥3), alcohol consumption (ever or never), and smoking status (ever or never). ^c^ Includes calcium from sources such as multivitamins, prenatal supplements, and calcium-specific supplements.

**Table 4:** Joint effects of vitamin D and calcium supplement use and odds of breast cancer.

Supplement Use	Cases/Controls	OR (95% CI) Basic Model ^a^	*p*	OR (95% CI) Multivariable Model ^b^	*p*
Never vitamin D user/never calcium user	43/76	Ref.	Ref.	Ref.	Ref.
Ever calcium use only	1/3	0.57 (0.06, 5.80)	0.64	0.80 (0.07, 9.24)	0.86
Ever vitamin D use only	1/7	0.24 (0.03, 2.04)	0.19	0.28 (0.03, 2.60)	0.26
Ever vitamin D use/ever calcium use	69/164	0.75 (0.47, 1.20)	0.24	0.60 (0.34, 1.03)	0.07

Unconditional logistic regression was used to estimate the OR and 95% CI. ^a^ Adjusted for age (continuous) and *BRCA1* or *BRCA2* mutation type. ^b^ Adjusted for age (continuous), *BRCA1* or *BRCA2* mutation type, menopausal status (premenopausal or postmenopausal), BMI (continuous), parity (0, 1–2, ≥3), alcohol consumption (ever or never), and smoking status (ever or never).

## Data Availability

Data described in the manuscript will not be made available.
